# Case Report: Cardiac metastasis of sarcomatoid urothelial carcinoma

**DOI:** 10.3389/fonc.2026.1763282

**Published:** 2026-05-12

**Authors:** Nan Ma, Jing Wang, Miaomiao Ma, Liping Yang

**Affiliations:** 1 Department of Cardiology (Ward 1), Gansu Provincial People’s Hospital, Lanzhou, China; 2 Department of Ultrasound Medicine, Gansu Provincial People’s Hospital, Lanzhou, China; 3 Department of Pathology, Gansu Provincial Cancer Hospital, Lanzhou, China; 4 Research Ward, Gansu Provincial People’s Hospital, Lanzhou, China

**Keywords:** cardiac metastasis, immunotherapy, PD-L1, right ventricular mass, sarcomatoid urothelial carcinoma, tislelizumab

## Abstract

**Background:**

Sarcomatoid urothelial carcinoma (SUC) is a rare, highly aggressive subtype of urothelial carcinoma (UC) accounting for fewer than 1%–2% of all urothelial malignancies. It is characterized by biphasic histology, a dismal prognosis, resistance to conventional chemotherapy, and a propensity for distant metastasis. Clinically confirmed cardiac metastasis from UC is exceedingly rare, with only sporadic cases documented worldwide.

**Case summary:**

We report a 55-year-old woman who presented with right-sided flank pain and gross hematuria. Percutaneous renal biopsy confirmed right renal pelvis SUC with approximately 85% sarcomatoid component. The patient underwent palliative robot-assisted laparoscopic nephroureterectomy; final pathological staging was pT3N1M1 (Stage IV). Approximately two months postoperatively, imaging confirmed multifocal metastases involving the lungs, pleura, liver, and lymph nodes, and first-line chemo-immunotherapy was initiated with gemcitabine plus carboplatin plus nivolumab. Carboplatin was substituted for cisplatin because the patient was deemed cisplatin-ineligible owing to renal insufficiency, following a modified CheckMate 901 framework. After one cycle, grade IV myelosuppression (platelet nadir 28 × 10^9^/L) necessitated permanent discontinuation of cytotoxic agents. During myelosuppression recovery, the patient developed palpitations; echocardiography revealed a large, broad-based right ventricular (RV) mass (50 × 41 mm). Treatment was revised to tislelizumab monotherapy (200 mg, every three weeks [q3w]). Serial echocardiographic follow-up documented progressive RV mass regression from 50 × 41 mm to 22 × 32 mm and ultimately to 11 × 14 mm, with stable systemic disease throughout.

**Conclusion:**

To our knowledge, this is the first documented case of isolated RV metastasis from right renal pelvis SUC achieving an objective response to anti-PD-1 immunotherapy. The sustained imaging regression under tislelizumab monotherapy strongly suggests—but does not definitively confirm—a metastatic etiology. For patients with high PD-L1–expressing SUC and cardiac metastasis who are intolerant of combined chemo-immunotherapy regimens, immune checkpoint inhibitor monotherapy may represent a feasible and well-tolerated treatment strategy.

## 1 Introduction

Sarcomatoid urothelial carcinoma (SUC) is an uncommon subtype of urothelial carcinoma (UC), comprising fewer than 1%–2% of all urothelial malignancies (1, 2). Histologically, SUC exhibits a biphasic architecture with both malignant epithelial and sarcomatoid stromal components, and it pursues a characteristically aggressive clinical course marked by rapid progression, early distant dissemination, and a short overall survival. Metastasis to the lungs, liver, bones, and regional lymph nodes is well recognized; however, cardiac involvement is exceptionally rare. Across all solid tumors, the prevalence of cardiac metastasis in autopsy series is estimated at 1.5%–3.5%, and clinically confirmed cardiac metastasis from UC remains an uncommon occurrence (1, 2).

Here we describe a patient with right renal pelvis SUC who developed a large, isolated RV mass consistent with cardiac metastasis during systemic treatment. Switching to tislelizumab monotherapy resulted in sustained regression of the RV mass. This case illustrates the rapidly progressive nature of SUC and the clinically occult presentation of intracardiac metastasis, and raises the possibility that single-agent PD-1 inhibition may be an effective and well-tolerated option for PD-L1–high SUC patients who cannot tolerate combined chemo-immunotherapy.

## 2 Case description

### 2.1 Patient background and social history

The patient was a 55-year-old woman with no history of tobacco use, no family history of urological malignancy, no known hereditary conditions, no prior oncological diagnoses or previous chemo/radiotherapy, and no regular medication use.

### 2.2 Clinical course and management

**Figure 1:**
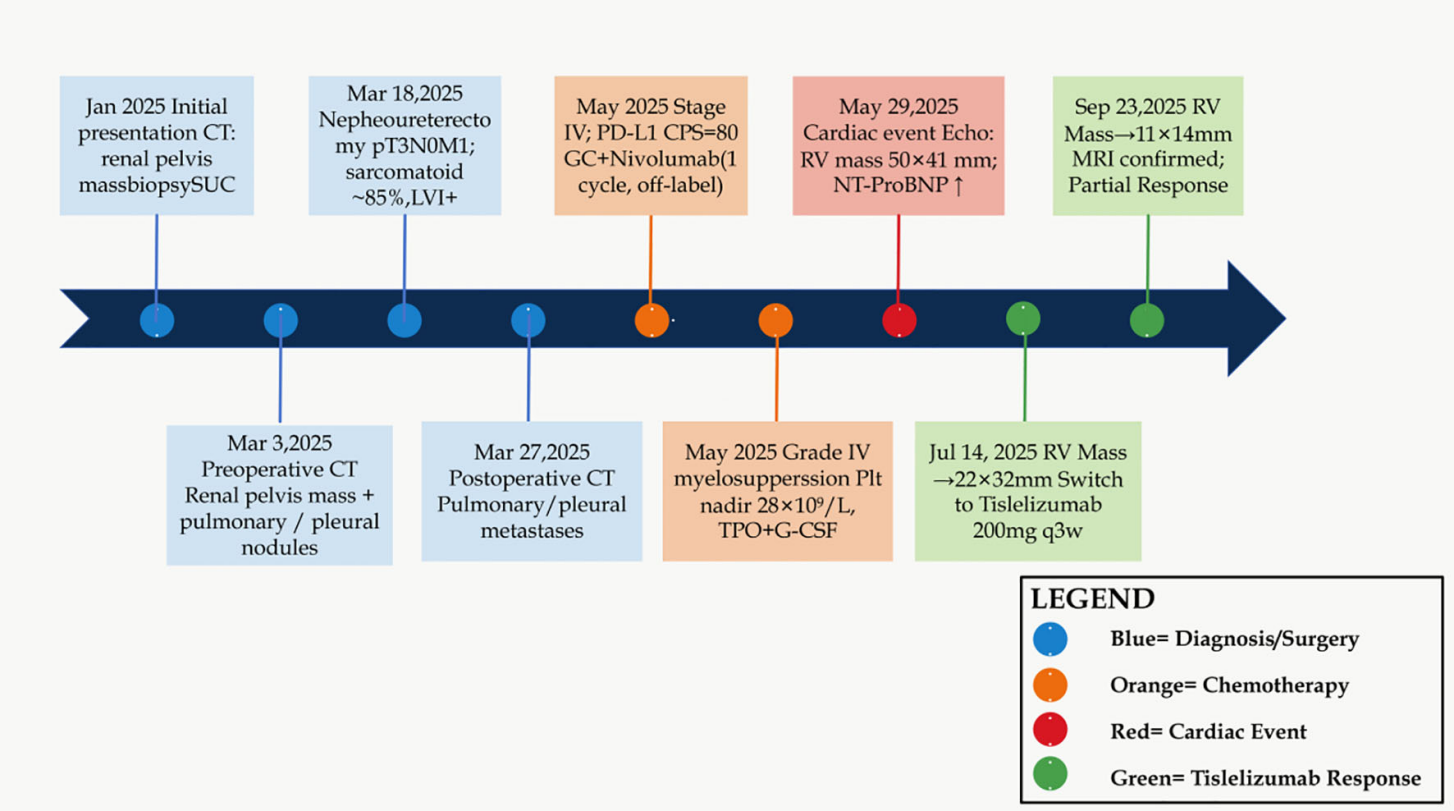
Clinical timeline of diagnosis, treatment, and cardiac metastasis response. Blue nodes: diagnosis and surgery; orange nodes: chemo-immunotherapy and hematological toxicity; red nodes: cardiac event; green nodes: tislelizumab monotherapy and tumor regression. SUC, sarcomatoid urothelial carcinoma; LVI, lymphovascular invasion; CPS, Combined Positive Score; GC, gemcitabine-carboplatin; TPO, thrombopoietin; G-CSF, granulocyte colony-stimulating factor; RV, right ventricle; MRI, magnetic resonance imaging.

The complete clinical timeline is summarized in **Figure 1**.

#### 2.2.1 January 2025 — initial presentation

**Figure 2:**
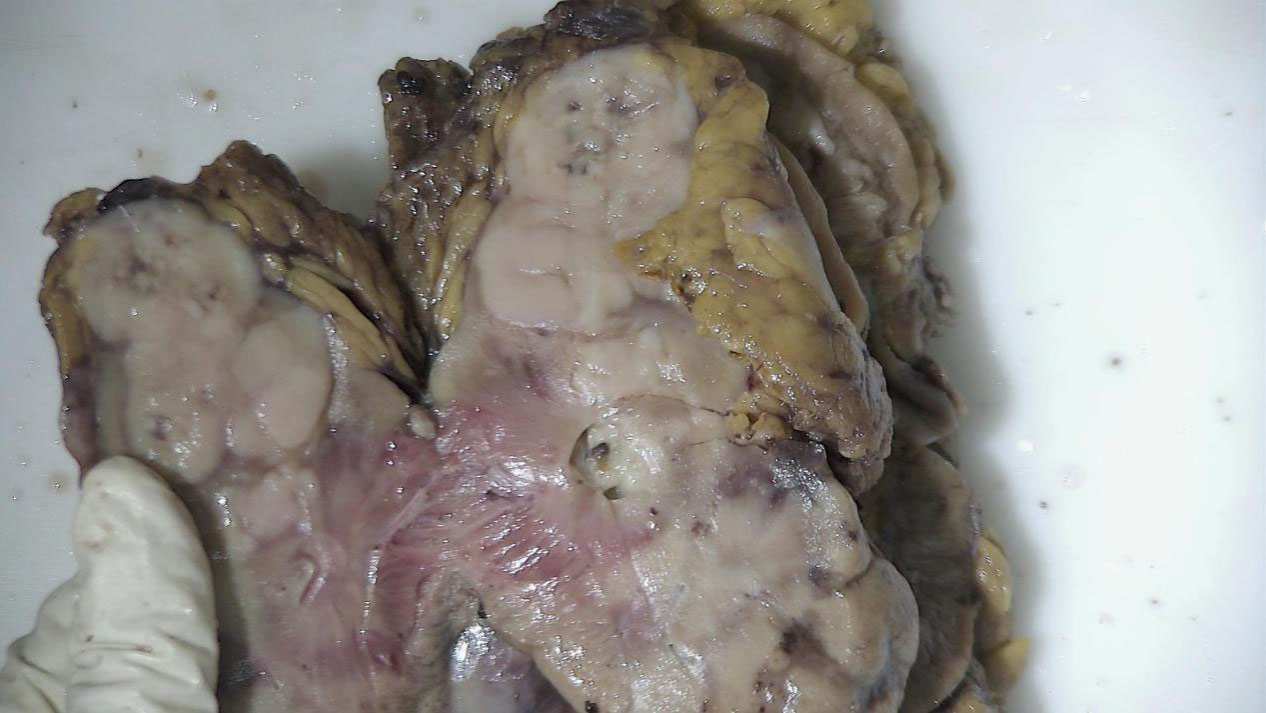
Gross specimen of the tumor resected during surgery.

**Figure 3:**
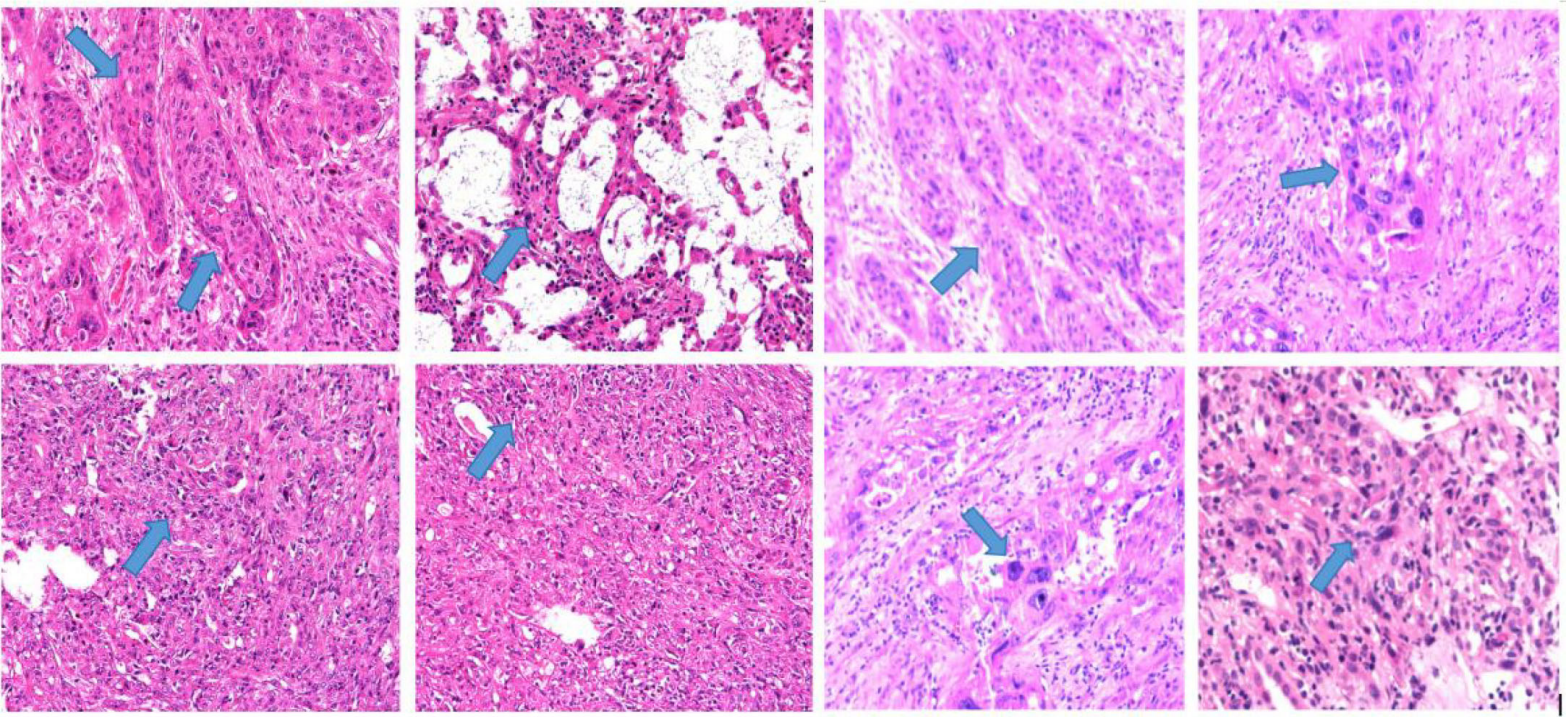
Histopathological findings from percutaneous renal biopsy, consistent with a malignant spindle-cell tumor suggestive of sarcomatoid urothelial carcinoma.

**Figure 4:**
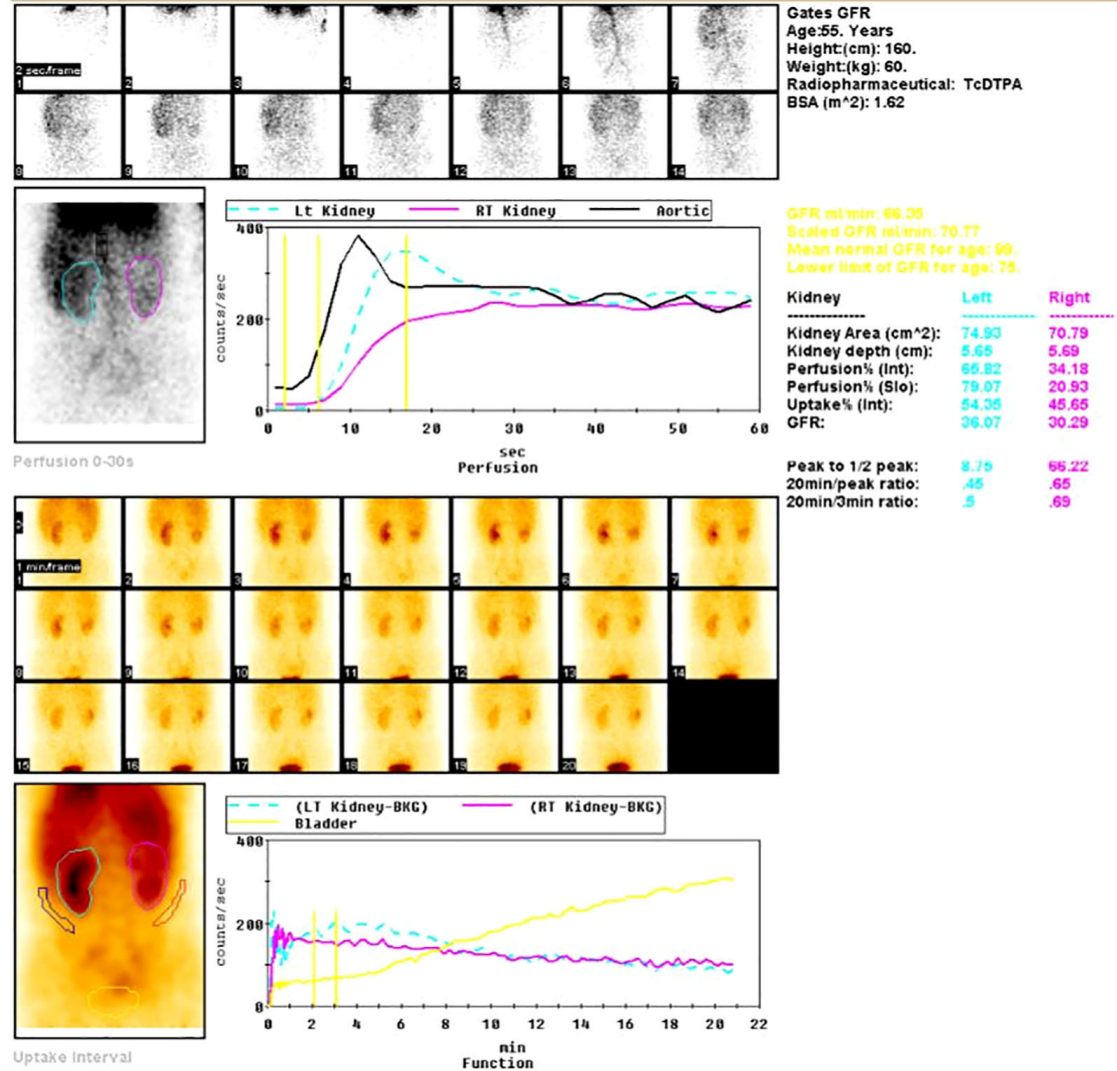
Renal ECT scan demonstrating normal perfusion with mild reduction in glomerular filtration rate and a space-occupying lesion in the right upper pole.

The patient presented to Gansu Provincial People’s Hospital with right-sided flank and back pain accompanied by nausea and vomiting. Self-administered levofloxacin and traditional herbal preparations failed to resolve her symptoms, after which gross hematuria developed, prompting further evaluation. Contrast-enhanced urinary tract computed tomography (CT) on 3 March 2025 revealed a right renal pelvis occupying lesion suspicious for malignancy, enlarged lymph nodes in the right renal region, multiple cysts in the left kidney, and a small volume of pelvic fluid (**Figure 2**). Percutaneous right renal biopsy identified a spindle-cell malignant neoplasm that, in conjunction with immunohistochemistry (IHC), was consistent with SUC (**Figure 3**). Concurrent renal radionuclide scintigraphy demonstrated mildly reduced bilateral glomerular filtration rates (GFR; left kidney 36.06 mL/min, right kidney 30.29 mL/min) and a photopenic defect in the upper right kidney consistent with the known mass (**Figure 4**).

#### 2.2.2 18 March 2025 — surgery

The patient underwent robot-assisted laparoscopic right nephroureterectomy with bladder cuff excision under general anesthesia. The procedure was uneventful. Intraoperative findings confirmed tumor involvement of the renal parenchyma and renal pelvis; the renal capsule and ureteral margin were free of tumor (**Figure 2**). In view of bilateral pulmonary and pleural nodules already apparent on preoperative CT, the intent of surgery was palliative resection.

#### 2.2.3 27 march 2025 — postoperative imaging

Chest-abdomen-pelvis and urinary tract CT demonstrated a small post-surgical fluid collection; a soft-tissue nodule along the course of the right renal vein (short-axis diameter approximately 19 mm, consistent with a metastatic lymph node); and multiple bilateral pulmonary and pleural nodules (largest 12 mm in the anterior segment of the right upper lobe).

#### 2.2.4 Final surgical pathology

The right nephroureterectomy specimen showed a high-grade mixed invasive urothelial carcinoma of the renal pelvis, predominantly sarcomatoid subtype (approximately 85%) with a conventional component of approximately 15%. Additional features included extensive tumor necrosis, a background of urothelial carcinoma *in situ*, invasion of perinephric adipose tissue and renal parenchyma, lymphovascular invasion (LVI), absence of perineural invasion, and a negative ureteral margin. IHC performed at the originating institution: CKpan (focally positive), Vimentin (focally positive), CK7 (small focus positive), CK20 (negative), P63 (small focus positive), GATA3 (focal weak positive), Uroplakin I (negative), PAX8 (negative), CA9 (negative), CD10 (focal positive), RCC (negative), AR (negative), Desmin (focal positive), SMA (negative), CD34 (negative), S-100 (negative), TLE1 (negative), SS18-SSX (negative), PSA (negative), P53 (negative, consistent with mutant pattern), Ki-67 (approximately 60%). Additional IHC performed at Peking University Cancer Hospital: HER2 (score 0, negative); PD-L1 (22C3 pharmDx assay) Combined Positive Score (CPS) = 80.

#### 2.2.5 May 2025 — first-line systemic therapy

Approximately two months postoperatively, restaging at Peking University Cancer Hospital confirmed Stage IV disease (pT3N1M1) with multifocal metastases (lymph nodes, bilateral lungs, pleura, liver). The patient was deemed cisplatin-ineligible on the basis of borderline bilateral renal function (total GFR < 70 mL/min) and her post-nephrectomy solitary-kidney status. In view of the exceptionally high PD-L1 CPS of 80, the multidisciplinary team initiated a carboplatin-substituted chemo-immunotherapy regimen: gemcitabine 1.5 g (days 1 and 8) plus carboplatin 0.4 g (day 1) plus nivolumab 360 mg (day 1), q3w — an off-label modification of the cisplatin-ineligible arm of the CheckMate 901 framework (nivolumab received regulatory approval in China in November 2024 for this indication). After the first cycle, the patient experienced grade IV myelosuppression: platelet nadir of 28 × 10^9^/L with concurrent grade III–IV leucopenia and neutropenia. Recombinant human thrombopoietin and granulocyte colony-stimulating factor (G-CSF) were administered; blood count recovery was achieved without transfusion, leading to permanent discontinuation of cytotoxic agents.

#### 2.2.6 29 May 2025 — cardiac event (during myelosuppression recovery)

**Figure 5:**
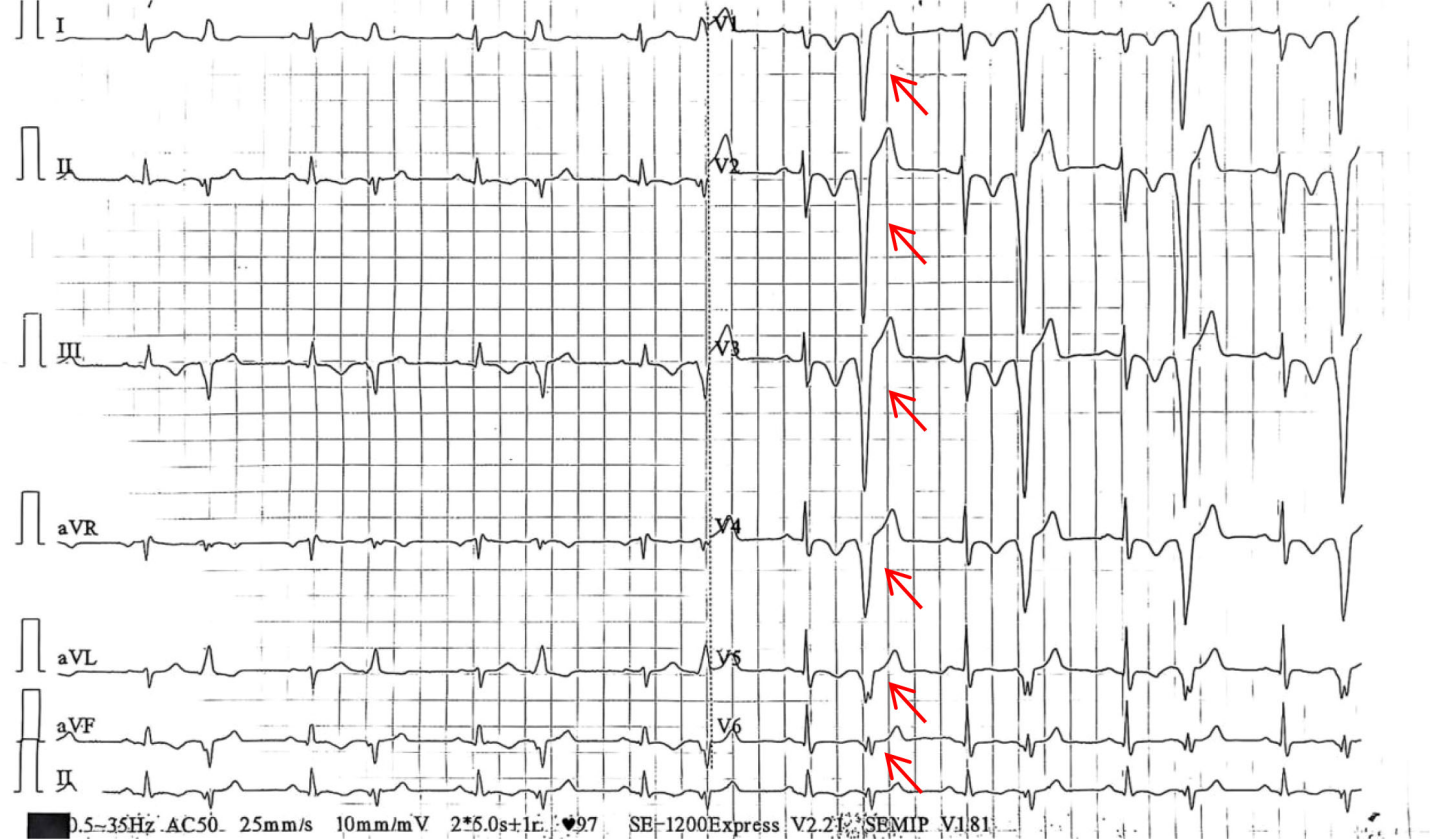
Standard electrocardiogram showing frequent premature ventricular contractions and ventricular bigeminy.

Approximately two weeks after cycle 1 administration and during hematological recovery, the patient developed acute-onset palpitations. Transthoracic echocardiography revealed, for the first time, a broad-based intracavitary RV mass (50 × 41 mm) with internal color Doppler flow signals and normal wall motion. A 12-lead electrocardiogram showed frequent premature ventricular contractions with ventricular bigeminy (**Figure 5**). NT-proBNP was elevated at 2965.8 pg/mL. Symptoms resolved spontaneously within 24 hours. A clinical diagnosis of cardiac metastasis was made. Endomyocardial biopsy was deferred owing to severe thrombocytopenia and the associated high hemorrhagic risk; the patient subsequently declined biopsy even after hematological recovery. Detailed diagnostic reasoning is presented in Section 3.2.

#### 2.2.7 19 July 2025 — treatment modification

Repeat echocardiography showed regression of the RV mass to 22 × 32 mm. Response was assessed as partial response. Given the intolerable hematological toxicity of cytotoxic therapy and the high PD-L1 CPS of 80, treatment was revised to tislelizumab 200 mg monotherapy, q3w. The spontaneous partial regression observed prior to initiation of tislelizumab may reflect a delayed immunological effect of the single dose of nivolumab administered. No further cytotoxic chemotherapy or nivolumab was administered at any point after the discovery of the cardiac mass; all subsequent systemic therapy consisted exclusively of tislelizumab monotherapy.

#### 2.2.8 23 September 2025 — follow-up

**Figure 6:**
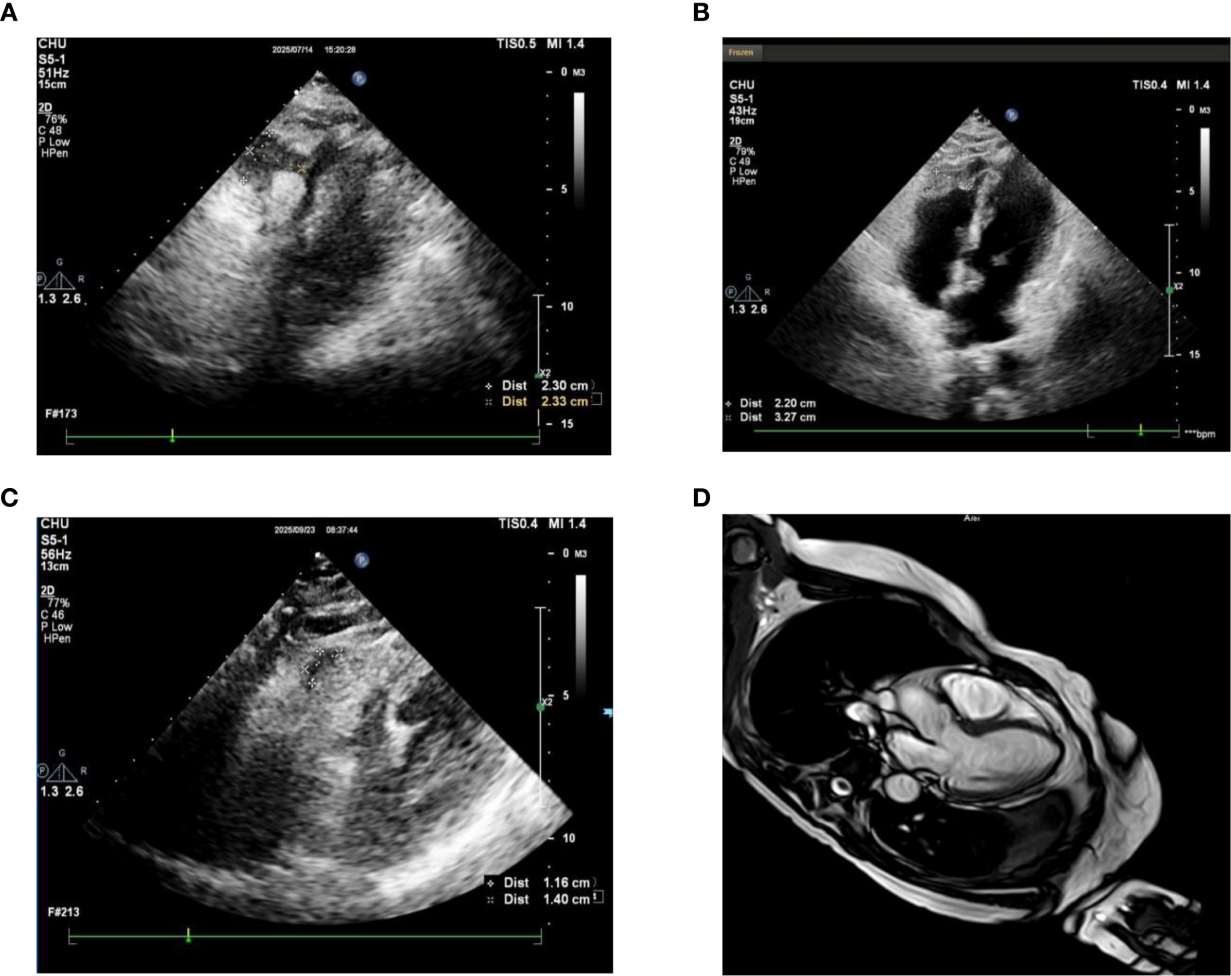
Follow-up cardiac imaging: transthoracic echocardiography and cardiac MRI demonstrating progressive shrinkage of the right ventricular mass. **(a)** Transthoracic echocardiography on 19 July 2025, showing the right ventricular mass. **(b)** Transthoracic echocardiography on 19 July 2025, showing the right ventricular mass, comparable to the July 14 image. **(c)** Transthoracic echocardiography on September 23, 2025, demonstrating marked reduction of the right ventricular mass. **(d)** Cardiac MRI showing the right ventricular mass on September 23, 2025.

The patient received tislelizumab infusions on schedule every 21–24 days. Repeat echocardiography demonstrated further regression of the RV mass to 11 × 14 mm. Cardiac magnetic resonance imaging (MRI) confirmed the broad-based right ventricular lesion (**Figure 6**). One episode of grade III myelosuppression occurred during this period and resolved with supportive treatment. At the last follow-up, systemic disease remained stable and no cardiac symptoms had recurred.

### 2.3 Physical examination

Breath sounds were clear bilaterally with no crackles or wheezes. Heart sounds were strong and regular, with no murmurs auscultated over any valve area. The abdomen was soft with no tenderness, rebound tenderness, or palpable masses. Costovertebral angle tenderness was absent bilaterally. Suprapubic tenderness was present without a palpable mass.

## 3 Discussion

### 3.1 Incidence and metastatic pathways of cardiac metastasis in urothelial carcinoma and sarcomatoid variants

Cardiac metastasis is identified in 1.5%–12% of solid tumor autopsies, yet clinical diagnosis remains infrequent (1). Within UC, cardiac involvement has been reported only in isolated cases. Pouwels et al. (1) described a complex case of bladder-origin SUC simultaneously complicated by sepsis and RV metastasis; Grech et al. (2) reported multiple cardiac metastases from conventional UC. Both cases underscore the predilection of urothelial tumors for right-sided cardiac chambers via the systemic venous circulation. To our knowledge, the present case is the first report of isolated RV metastasis from right renal pelvis SUC with an objective, treatment-attributable radiological response.

The most plausible metastatic route is hematogenous spread via the renal vein → inferior vena cava → right atrium → right ventricle, analogous to the mechanism of inferior vena cava tumor thrombus extension in renal cell carcinoma (3). Despite a maximum mass dimension of 50 × 41 mm, the patient experienced only transient palpitations, further reinforcing the well-recognized clinical silence of intracardiac metastatic disease.

### 3.2 Diagnostic challenges: absence of histological confirmation and differential diagnosis

A significant limitation of this case is the absence of pathological confirmation of the intracardiac lesion. At initial presentation, grade IV thrombocytopenia precluded percutaneous endomyocardial biopsy owing to prohibitive hemorrhagic risk; once hematological recovery had occurred, the patient declined biopsy in view of the ongoing mass regression. The diagnosis therefore rested on clinical and imaging inference.

The differential diagnosis encompasses primary cardiac tumors. Atrial myxoma typically originates in the left atrium on a stalk and does not respond to immunotherapy. Primary cardiac sarcoma may arise in the right ventricle but would similarly not be expected to regress with PD-1 blockade. Four lines of indirect evidence support a metastatic a etiology: (1) the primary SUC demonstrated pT3 disease with LVI; (2) multifocal distant metastases were confirmed concurrently with the discovery of the cardiac mass; (3) the timing of RV mass regression correlated closely with immunotherapy administration; and (4) cardiac MRI demonstrated a broad-based, sessile morphology consistent with metastatic disease rather than a pedunculated primary tumor. All conclusions regarding treatment response are characterized as strongly suggestive rather than definitively proven; future cases should pursue pathological confirmation whenever clinically feasible.

### 3.3 Immunobiology of SUC: mechanistic basis for immune checkpoint inhibitor sensitivity

Sarcomatoid differentiation in UC represents the terminal common pathway of epithelial–mesenchymal transition (EMT)-driven dedifferentiation (4). While conferring tumor invasiveness, this process paradoxically remodels the tumor microenvironment (TME) towards a state of heightened immunotherapy sensitivity. Recent case series of upper tract urothelial carcinoma (UTUC) with sarcomatoid differentiation have reported PD-L1 CPS values of 30–90 (5), substantially higher than those observed in conventional UC. Digital spatial transcriptomic analyzes have demonstrated that sarcomatoid regions are enriched for CD163-positive M2-polarised tumor-associated macrophages and activated cancer-associated fibroblasts (6), which drive PD-L1 upregulation through IFN-γ and TGF-β signaling. This immunosuppressive infiltrate, which attenuates antitumor immunity prior to treatment, becomes a highly responsive substrate once PD-1/PD-L1 blockade is applied. This mechanism provides a biological rationale for the marked response to tislelizumab observed in our patient despite her inability to tolerate cytotoxic agents.

A further question worth addressing is whether the single dose of nivolumab exerted an immune-priming effect that potentiated the subsequent response to tislelizumab. The spontaneous regression of the RV mass from 50 × 41 mm to 22 × 32 mm during the treatment-free interval between 29 May and 19 July 2025 may reflect a delayed nivolumab effect consistent with the immune memory properties of checkpoint inhibitors. This hypothesis remains unproven but should be acknowledged when interpreting the magnitude of the tislelizumab response.

### 3.4 Therapeutic rationale, guidelines, and the role of tislelizumab monotherapy

No standardized treatment regimen currently exists for metastatic SUC. Current ESMO/EAU guidelines and the CheckMate 901 trial support gemcitabine-cisplatin-nivolumab as first-line therapy for cisplatin-eligible Stage IV UC (7). The carboplatin-substituted modification employed in this case for a cisplatin-ineligible patient remains an off-label adaptation, and the resultant grade IV myelosuppression ultimately necessitated permanent cessation of cytotoxic therapy.

For cisplatin-ineligible patients with high PD-L1 expression, enfortumab vedotin plus pembrolizumab (EV-302/KEYNOTE-A39) currently represents the preferred standard of care. However, this combination was not administered in the present case owing to concerns regarding peripheral neurotoxicity and institutional accessibility constraints. The choice of tislelizumab monotherapy was based on its demonstrated phase II efficacy in previously treated metastatic UC (objective response rate approximately 22%) (8), as well as its Fc-region engineering, which eliminates antibody-dependent cellular phagocytosis (ADCP) and theoretically sustains more durable T-cell activation.

The regression of the RV mass from 50 × 41 mm to 11 × 14 mm over approximately four months of tislelizumab treatment represents a clinically meaningful, imaging-documented treatment response. Although the absence of pathological confirmation precluded formal RECIST evaluation of the cardiac lesion, serial echocardiographic and MRI measurements provide compelling longitudinal evidence of treatment activity. The observed regression spans both the nivolumab-priming period and the tislelizumab treatment period, and attribution between the two agents remains unresolvable. Analogous responses to single-agent PD-1 inhibition have been observed in sarcomatoid renal cell carcinoma and sarcomatoid non-small-cell lung cancer (5), lending support to the concept of sarcomatoid differentiation as a broad cross-tumor predictor of immunotherapy sensitivity.

### 3.5 Strengths and limitations

#### 3.5.1 Limitations

(1) Pathological confirmation of the intracardiac lesion was not obtained; all conclusions regarding treatment response should be interpreted as strongly suggestive rather than definitively established. (2) The follow-up duration is relatively short, and long-term durability of immunotherapy benefit remains to be determined. (3) A primary right ventricular sarcoma cannot be entirely excluded on imaging alone. (4) The substitution of carboplatin for cisplatin complicates interpretation of the grade IV myelosuppression event. (5) Attribution of efficacy between the single dose of nivolumab and subsequent tislelizumab therapy cannot be resolved in a single case.

#### 3.5.2 Strengths

(1) To our knowledge, this report constitutes the first documented case of isolated RV metastasis from right renal pelvis SUC achieving an objective response to anti-PD-1 therapy. (2) Serial quantitative echocardiographic and MRI measurements provide an objective longitudinal dataset of intracardiac tumor behavior under systemic immunotherapy. (3) A PD-L1 CPS of 80 situates this case within an emerging mechanistic framework linking EMT-driven PD-L1 upregulation in sarcomatoid tumors to immunotherapy sensitivity.

## 4 Patient perspective

The patient remained cooperative and engaged throughout the evaluation and treatment process. Following regression of the right ventricular mass and resolution of cardiac symptoms, her quality of life improved progressively. She expressed relief at the response to immunotherapy and stated her firm intention to continue treatment and long-term follow-up. Her adherence to scheduled clinic visits was excellent throughout the follow-up period.

## Data Availability

The raw data supporting the conclusions of this article will be made available by the authors, without undue reservation.
